# Biofilms and antibiotic susceptibility of multidrug-resistant bacteria from wild animals

**DOI:** 10.7717/peerj.4974

**Published:** 2018-06-12

**Authors:** Carla Dias, Anabela Borges, Diana Oliveira, Antonio Martinez-Murcia, Maria José Saavedra, Manuel Simões

**Affiliations:** 1 LEPABE-Department of Chemical Engineering, Faculty of Engineering—University of Porto, Portugal; 2 CITAB, Centre for the Research and Technology of Agro-Environment and Biological Sciences, Universidade de Tras-os-Montes e Alto Douro, Portugal; 3 CECAV, Veterinary and Animal Science Research Center, Universidade de Tras-os-Montes e Alto Douro, Portugal; 4 CIQUP, Department of Chemistry and Biochemistry, Faculty of Sciences, University of Porto, Portugal; 5 Area of Microbiology, EPSO, University Miguel Hernández, Alicante, Spain

**Keywords:** Biofilms, Ciprofloxacin, Imipenem, Wild animals, Multidrug-resistance, β-lactamases

## Abstract

**Background:**

The “One Health” concept recognizes that human health and animal health are interdependent and bound to the health of the ecosystem in which they (co)exist. This interconnection favors the transmission of bacteria and other infectious agents as well as the flow of genetic elements containing antibiotic resistance genes. This problem is worsened when pathogenic bacteria have the ability to establish as biofilms. Therefore, it is important to understand the characteristics and behaviour of microorganisms in both planktonic and biofilms states from the most diverse environmental niches to mitigate the emergence and dissemination of resistance.

**Methods:**

The purpose of this work was to assess the antibiotic susceptibility of four bacteria (*Acinetobacter* spp., *Klebsiella pneumoniae*, *Pseudomonas fluorescens* and *Shewanella putrefaciens*) isolated from wild animals and their ability to form biofilms. The effect of two antibiotics, imipenem (IPM) and ciprofloxacin (CIP), on biofilm removal was also assessed. Screening of resistance genetic determinants was performed by PCR. Biofilm tests were performed by a modified microtiter plate method. Bacterial surface hydrophobicity was determined by sessile drop contact angles.

**Results:**

The susceptibility profile classified the bacteria as multidrug-resistant. Three genes coding for β-lactamases were detected in *K. pneumoniae* (TEM, SHV, OXA-aer) and one in *P. fluorescens* (OXA-aer). *K. pneumoniae* was the microorganism that carried more β-lactamase genes and it was the most proficient biofilm producer, while *P. fluorescens* demonstrated the highest adhesion ability. Antibiotics at their MIC, 5 × MIC and 10 × MIC were ineffective in total biofilm removal. The highest biomass reductions were found with IPM (54% at 10 × MIC) against *K. pneumoniae* biofilms and with CIP (40% at 10 × MIC) against *P. fluorescens* biofilms.

**Discussion:**

The results highlight wildlife as important host reservoirs and vectors for the spread of multidrug-resistant bacteria and genetic determinants of resistance. The ability of these bacteria to form biofilms should increase their persistence.

## Introduction

Every year tons of antibiotic residues are discarded into natural resources, making the environment a reservoir of bacteria carrying resistance genes (Davies & Davies, 2010; Fukuda et al., 2016). Moreover, antibiotic resistance is a natural phenomenon in that the microorganisms from the environment and human pathogens share the same resistome (Dantas et al., 2008; Forsberg et al., 2012; Kraemer et al., 2017). In recent years it has been observed that a drastic increase of pathogenic bacterial strains resistant to multiple antibiotics, including *Klebsiella pneumoniae*, *Acinetobacter baumannii*, *Pseudomonas aeruginosa* and other Gram-negative pathogenic species (Finley et al., 2013; Moyaert et al., 2017).  In Gram-negative bacteria, one of the most important mechanisms of antibiotic resistance is the production of β-lactamases (Hawkey & Jones, 2009). These enzymes are usually acquired by horizontal gene transfer and confer resistance to β-lactams, the most commonly used class of antibiotics for the treatment of human and animal infections (Henriques et al., 2006). Another major factor contributing to antibiotic resistance is the ability of microorganisms to form sessile communities on both biotic and abiotic surfaces (Høiby et al., 2010). Biofilms can be defined as a dynamic biological system of microbial cells that are strongly associated with a surface and embedded in an organic polymeric matrix of microbial origin. It is estimated that more than 65% of microbial infections are caused by microorganisms when they grow in biofilms (Cook & Dunny, 2014). It is known that bacterial cells during their transition from planktonic to sessile state undergo extensive changes (e.g., behaviour, structure and physiology) which are reflected in the phenotypic and metabolic characteristics of biofilm cells (O’Toole, Kaplan & Kolter, 2000). Resistance mechanisms described for planktonic cells such as antibiotic modifying enzymes, target modification, and efflux pumps cannot solely explain the high resistance of biofilm cells (Hughes & Andersson, 2017; Penesyan, Gillings & Paulsen, 2015; Vanegas et al., 2009). In fact, the mechanisms of antibiotic resistance in biofilms are not fully understood (Mah & O’Toole, 2001). Biofilm resistance is known to vary from one microorganism to another, being a combination of several mechanisms strongly influenced the environmental conditions (Anderson & O’Toole, 2008; Dufour, Leung & Lévesque, 2010).

The World Health Organization recently recognised antibiotic resistance as a serious global problem, not only in terms of human health but also for the animals (both domestic and wildlife) and the environment (Gibbs, 2014). Indeed, some bacteria can spread between different ecosystems, from animals and humans to water and soil. Besides, the exchange of resistance genes among bacterial strains from different environments can also occur, suggesting that when resistance arises it is not restrained to the niche where it first appeared  (Da Costa, Loureiro & Matos, 2013). Several studies have demonstrated that antibiotic-resistant strains are present in a wide variety of ecological niches, including wild bird species that inhabit in remote ecosystems (Smith et al., 2014; Freitas et al., 2018). However, the knowledge on the propensity of these strains to establish sessile communities and on the consequent advantages to survive under adverse conditions is scarce (Davies & Davies, 2010). This study characterizes phenotypically and genotypically the antibiotic resistance pattern of four bacterial strains (*Acinetobacter* spp., *Klebsiella pneumoniae*, *Pseudomonas fluorescens* and *Shewanella putrefaciens*) isolated from faecal samples of different species of wild animals. These bacteria were selected from 370 different isolates based on their potential threat for public health and due to their resistance to imipenem (IPM), an antibiotic restricted for hospital use (Dias et al., 2014). The ability of the selected bacteria to form biofilms *in vitro* was assessed as well as the effects of the antibiotics ciprofloxacin (CIP) and IPM on biofilm removal. The relationship between antibiotic resistance, the presence of resistance genetic determinants and biofilm production was also assessed.

## Materials and Methods

### Sample collection and bacterial isolation

Bacterial isolates were collected aseptically from freshly voided faecal samples of different species of wild animals, including birds, reptiles and mammals, at the north of Portugal (Dias et al., 2014). Collection was done as soon as the animal entered the Centre for Treatment of Wild Animals (Veterinary Hospital of the University of Trás-os-Montes and Alto Douro). MacConkey agar (Merck, Germany) was used for bacterial isolation and the plates were incubated at 37 °C for 24 h. Afterwards, the pure cultures were checked for oxidase activity (1% tetramethyl phenylenediamine; Merck, Darmstadt, Germany) before cryopreservation in Brain Heart Infusion broth (BHI; Oxoid, Cheshire, UK) with 30% (v/v) glycerol at −70 °C.

### Antibiotic susceptibility testing

The antimicrobial sensitivity profile of recovered bacteria was determined using the disk diffusion assay on Muller-Hinton agar (MH; Oxoid, Cheshire, UK) according to the standards and interpretive criteria described by the Clinical and Laboratory Standards Institute guidelines (CLSI, 2014). The strains were tested for susceptibility to a panel of antibiotics (Oxoid, UK): amoxicillin (AML_10_), amoxicillin/clavulanic acid (AMC_30_), ticarcillin (TIC_75_), ticarcillin/clavulanic acid (TIM_85_), piperacillin (PRL_100_), piperacillin/tazobactam (TZP_110_), cephalothin (KF_30_), cefoxitin (FOX_30_), ceftriaxone (CRO_30_), cefoperazone (CFP_30_), ceftazidime (CAZ_30_), cefotaxime (CTX_30_), cefepime (FEP_30_), imipenem (IPM_10_), aztreonam (ATM_30_), streptomycin (S_10_), kanamycin (K_30_), amikacin (AK_30_), gentamicin (CN_10_), tobramycin (TOB_10_), nalidixic acid (NA_30_), ciprofloxacin (CIP_5_), erythromycin (E_15_), tetracycline (TE_30_), trimethoprim-sulfamethoxazole (SXT_25_), chloramphenicol (C_30_) and fosfomycin (FOS_50_).

### Identification of bacterial resistant isolates

Identification was performed by 16S rRNA gene sequence analysis. For that, general bacterial 5′ AGA GTT TGA TCA TGG CTC AG 3′ forward primer and 5′ GGT TAC CTT GTT ACG ACT T 3′ reverse primer were used to amplify nearly full-length 16S rRNA gene as described previously (Macfarlane et al., 2004; Soler et al., 2004). Sequencing analysis were performed using the BigDye Terminator V3.1 cycle sequencing kit ant the ABI Prism® 3100 Avant Genetic Analyzer (Applied Biosystems, Foster City, CA, USA). The nucleotide sequences were edited using the software Chromas 2 and compared with published sequences in the Nacional Center for Biotechnology Information (NCBI) databases using BLAST. Phylogenetic tree was produced by the neighbor-joining method (Saitou & Nei, 1987) with Kimura’s 2-parameter method (Kimura, 1980) using the MEGA4-molecular evolutionary genetic analysis program (Tamura et al., 2007).

### Minimal inhibitory concentration (MIC) determination

Standardized planktonic MIC’s of ciprofloxacin (CIP) (Sigma-Aldrich Co., Lisbon Portugal) and imipenem (IPM) (Cayman Chemical, Ann Arbor, MI, USA) were assessed by the microdilution method outlined by CLSI (2014). Overnight cell cultures (18 h incubation) were adjusted to a cell density of 1 × 10^6^ cells/ml and added to sterile 96-well polystyrene microtiter plates (Orange Scientific, USA) containing different concentrations (CIP-6, 8, 10, 14, 16, 24, 32, 36, 40, 44, 52, 56 and 60 µg/ml; IPM-2, 4, 6, 8, 10, 12 µg/ml) of each antibiotic in a final volume of 200 µl. A bacterial suspension without antibiotic was used as negative control. Microtiter plates were then incubated at 30 °C for 24 h. MIC matched to the lowest concentration in which the final optical density (OD) at 620 nm was equal or lower than the initial OD.

### Screening of resistance genetic determinants: *β*-lactamases

Detection of genetic determinants was done for TEM-type, SHV-type, CTX-M-type, MOX and FOX variants of extended-spectrum-β-lactamases (ESBLs) and for the CphA, IMP-type, VIM-type and OXA-type carbapenemases. The mixture of PCR was performed in a final volume of 50 µl containing 5 µl of Biotools PCR buffer (10×), 200 µM of each nucleotide, 10 pmol of each primer (Table 1), 1 µl (1U) of Taq DNA polymerase (Biotools) and 1 µl of template genomic DNA (100–200 ng). The conservative oligonucleotide primers used were developed by Fontes (2009). The amplification conditions were: initial denaturation for 5 min at 95 °C, 35 amplification cycles composed of a denaturation step for 15 s at 94 °C, an annealing step at 55 °C for 30 s, except for *bla*_FOX_, in which 60 °C were used, and an extension period at 72 °C for 45 s. The reaction was completed with an extension step at 72 °C for 3 min.

**Table 1:** Oligonucleotide primers used (Fontes, 2009).

**Target**	**Primer pair**	**Sequence 5′−3**	**Amplicon size (bp)**
*bla*_TEM_	TEM-F	CTG GAT CTC AAC AGC GGT AAG	403
	TEM-R	ACG TTG TTG CCA TTG CTG CAG	
*bla*_SHV_	SHV-F	TTC GCC TGT GTA TTA TCT CC	373
	SHV-R	GTT ATC GCT CAT GGT AAT GG	
*bla*_CTX-M_	CTX-BF	GCC TGC CGA TCT GGT TAA C	209
	CTX-BR	GGA ATG GCG GTA TTC AGC G	
*bla*_MOX_	MOX-F	CGT GCT CAA GGA TGG CAA G	634
	MOX-R	CTG CTG CAA CGC CTT GTC A	
*bla*_FOX_	FOX-F	TGT TCG AGA TTG GCT CGG TC	282
	FOX-R	GGG TTG GAA TAC TGG CGA TG	
*bla*_CphA_	CphA-3F	CTG GAG GTG ATC AAC ACC A	410
	CphA-7R	TTG ATC GGC AGC TTC ATC GC	
*bla*_VIM_	VIM-F	GGT GTT TGG TCG CAT ATC GC	195
	VIM-R	CAT GAA AGT GCG TGG AGA CTG	
*bla*_OXA_	OXA-aerF	GAC TAC GGC AAC CGG GAT C	215
	OXA-aerR	CTT GCC GTG GAT CTG CCA G	
	OXA-BF	GAT AGT TGT GGC AGA CGA ACG	453
	OXA-BR	CTT GAC CAA GCG CTG ATG TTC	
	OXA-CF	GTT CTC TGC CGA AGC CGT CA	554
	OXA-CR	GAC TCA GTT CCC ACA CCA G	
*bla*_IMP_	IMP-F	GAAGGCGTTTATGTTCATAC	559
	IMP-R	CTTCACTGTGACTTGGAAC	

### Bacterial surface hydrophobicity evaluation

Surface hydrophobicity was evaluated after contact angle measurement according to the procedure described by Simões, Simões & Vieira (2010). Lawns of each bacterium were prepared as described by Busscher et al. (1984) and their contact angles were determined by the sessile drop contact angle measurements using a model OCA 15Plus (DATAPHYSICS, Germany), with three pure liquids (water, formamide, and *α*-bromonaphthalene). The surface tension components of the three liquids were obtained from the literature (Janczuk et al., 1993). Afterwards, the hydrophobicity ($\Delta {G}_{bwb}^{TOT}$) and surface tension parameters (ϒ^*AB*^—Lewis acid–base component, ϒ^+^—electron acceptor parameter, ϒ^−^—electron donor parameter) of the strains were evaluated according to the method of Van Oss et al. (1989).

### Initial adhesion assay

*In vitro* initial adhesion was determined according to the method of Simões et al. (2007) and Simões, Simões & Vieira (2010). Sterile microtiter plates (12-well) with 2 ml of cell suspension (1 × 10^8^ cells/ml in MH) and coupons (dimensions of 1 × 1 cm) of polystyrene (PS) horizontally placed were incubated at 30 °C for 2 h in an orbital shaker at 150 rpm. PS coupons were prepared by successive immersions in a solution (5% v/v) of commercial detergent (Sonasol Pril; Henkel Ibérica S.A, Barcelona, Spain) and ultrapure water with gentle shaking (30 min). All experiments were performed in triplicate, with three independent repeats. After 2 h incubation the coupons were removed and washed in 2 ml of sterile saline solution (0.85% w/v) and the biomass was quantified by crystal violet (CV) staining (Borges et al., 2017).

### Biofilm formation

Biofilm formation was performed in 96-well polystyrene microplates following the method of Stepanović et al. (2000). Briefly, overnight cultures were adjusted to an initial OD (620 nm) of 1 × 10^8^ cells/ml in MH and 200 µl aliquots were added to the microplate. The microtiter plate was incubated for 24, 48 and 72 h at 30 °C and agitated at 150 rpm. The medium (MH) was carefully and aseptically replaced daily by fresh one, after a washing step with saline solution (0.85% v/v). Negative controls contained MH broth without bacteria.

### Biofilm control

To determine whether antibiotics had capability to remove 24 h old biofilms, CIP and IPM at MIC, 5 × MIC and 10 × MIC were used according to Borges et al. (2017). After 24 h exposure at 30 °C the antibiotic solutions were discarded and the biofilms were analyzed in terms of biomass by CV staining. The results are presented as percentage of biofilm mass removal when compared to biofilms non-exposed to antibiotics. The assays were performed in triplicate with three independent repeats.

### Mass quantification of adhered cells and biofilms

The mass of adhered cells and biofilm was quantified by CV staining according to Borges et al. (2017). The OD was measured at 570 nm using a Microplate Reader (BIO-TEK, Model Synergy HT). Biofilm mass removal percentage (%BR) was given by the following equation:

\begin{eqnarray*}\text{%}\mathrm{BR}= \frac{\mathrm{ODn}-\mathrm{ODw}}{\mathrm{ODn}} \times 100. \end{eqnarray*}

Where the ODn are the absorbance values of the biofilms non-exposed to the antibiotics (CIP and IPM) and ODw are the absorbance values of the biofilms exposed to antibiotics (CIP and IPM).

### Adherent/biofilm bacteria classification

Based on the cutoff of the ODc quantification (value defined as three standard deviation values above the mean OD of the negative control) the bacteria were classified according to Stepanović et al. (2000) as: non-adherent/non-biofilm producer (0)-OD ≤ ODc; weakly adherent/weak biofilm producer (+)-ODc < OD ≤ 2 × ODc; moderately adherent/moderate biofilm producer (++)-2 × ODc < OD ≤ 4 × ODc; strongly adherent/strong biofilm producer (+++)-4 × ODc < OD.

### Statistical analysis

Data were analyzed by analysis of variance (ANOVA) using the statistical program SPSS version 22.0 (SPSS, Inc., Chicago, IL, USA) and statistical calculations were based on a confidence level ≥ 95% (*P* < 0.05 was considered statistically significant).

## Results

### Bacteria and antibiotic susceptibility profile

The selected bacteria belong to different genera: *Acinetobacter*, *Klebsiella*, *Pseudomonas* and *Shewanella*. *S. putrefaciens* was obtained from a sample of red deer (*Cervus elaphus*), *P. fluorescens* from a red fox (*Vulpes vulpes*) and *K. pneumoniae* and *Acinetobacter* spp. were isolated from the same sample of a greater rhea (*Rhea americana*). These four strains were identified through 16S rDNA sequencing and were selected due to their antibiotic resistance profiles. *Acinetobacter* spp. was only identified at the genus level. According to the analysis of the phylogenetic tree generated (See Fig. S1), this strain can represent a new species.

The susceptibility profile against 27 antibiotics belonging to different classes was assessed. All bacteria were multidrug-resistant, i.e., resistant to at least two antibiotics belonging to different chemical classes (Godebo, Kibru & Tassew, 2013; Exner et al., 2017) (Table 2). It was possible to observe that *Acinetobacter* spp. and *K. pneumoniae*, showed identical resistance profiles. This could be related to the fact that these bacteria were isolated from the same sample, indicating an exchange of resistance genes. Among all the isolates, *S. putrefaciens* was the most susceptible to the antibiotics tested. All bacteria were resistant to amoxicillin, amoxicillin/clavulanic acid, cephalothin, fosfomycin and erythromycin. Of particular concern was the resistance of all the selected bacteria to the carbapenem imipenem (IPM), an intravenous broad-spectrum antibiotic of the β-lactams group that is of exclusive hospital use (Papp-Wallace et al., 2011).

**Table 2:** Resistance phenotypes and *bla* genotypes of the four selected bacteria.

**Strains identification (16S rRNA gene sequencing)**	**Resistance phenotype**	β-**lactamase genes**
*Acinetobacter* spp.	AML, AMC, TIM, ATM, IPM, KF, FOS, FOX, CFP, CIP, S, E, C, SxT, TE	–
*K. pneumoniae*	AML, AMC, TIM, ATM, IPM, KF, FOS, FOX, CFP, CIP, S, E, C, SxT, TE	*bla*_OXA-aer_; *bla*_TEM_; *bla*_SHV_
*P. fluorescens*	AML, AMC, TIC, TIM, ATM, IPM, KF, FOS, FOX, CTX, E, C, SxT, TE	*bla*_OXA-aer_
*S. putrefaciens*	AML,AMC, IPM, KF, FOS, E	–

**Notes.**

Amoxicillin (AML_10_), amoxicillin/clavulanic acid (AMC_30_), ticarcillin (TIC_75_), ticarcillin/clavulanic acid (TIM_85_), piperacillin (PRL_100_), piperacillin/tazobactam (TZP_110_), cephalothin (KF_30_), cefoxitin (FOX_30_), ceftriaxone (CRO_30_), cefoperazone (CFP_30_), ceftazidime (CAZ_30_), cefotaxime (CTX_30_), cefepime (FEP_30_), imipenem (IPM_10_), aztreonam (ATM_30_), streptomycin (S_10_), kanamycin (K_30_), amikacin (AK_30_), gentamicin (CN_10_), tobramycin (TOB_10_), nalidixic acid (NA_30_), ciprofloxacin (CIP_5_), erythromycin (E_15_), tetracycline (TE_30_), trimethoprim-sulfamethoxazole (SXT_25_), chloramphenicol (C_30_) and fosfomycin (FOS_50_).

### Genetic bases of antibiotic resistance

The presence of 12 β-lactamase genes (*bla*_CphA_; *bla*_OXA-aer_; *bla*_OXA-B_; *bla*_OXA-C_; *bla*_MOX_; *bla*_TEM_; *bla*_FOX_; *bla*_SHV_; *bla*_SHV_; *bla*_CTX-M_; *bla*_IMP_ and *bla*_VIM_) in the selected bacteria was analyzed by PCR. The genotype results are summarized in Table 2. The *bla*_OXA-aer_ was detected in *P. fluorescens* and *K. pneumoniae*, isolated from faecal samples of a fox and a rhea, respectively. Three β-lactamases genes (*bla*_OXA-aer_; *bla*_TEM_; *bla*_VIM_) were detected in the same bacterium, *K. pneumoniae*. *S. putrefaciens* and *Acinetobacter* spp. were resistant to IPM but apparently did not display any of the genetic determinants of IPM resistance studied in this work. No PCR specific *bla*_CphA_; *bla*_OXA-B_; *bla*_OXA-C_; *bla*_MOX_; *bla*_FOX_; *bla*_SHV_; *bla*_CTX-M_; *bla*_IMP_ and *bla*_VIM_ encoding sequences were detected.

### Minimal inhibitory concentration of ciprofloxacin and imipenem

*K. pneumoniae* was the bacterium with lowest susceptibility to the action of CIP with MIC of 60 µg/ml (Table 3). *S. putrefaciens* was the most resistant to IPM with MIC of 12 µg/ml. In general, IPM was the most efficient antibiotic against the four bacteria tested (MICs: 12 µg/ml for *S. putrefaciens*; 6 µg/ml for *P. fluorescens*, 6 µg/ml for *K. pneumonia*; 2 µg/ml for *Acinetobacter* spp.). According to the CLSI (2014) guidelines all isolates are considered resistant to CIP (MICs > 4 µg/ml). *K. pneumonia* ***,*** *P. fluorescens* and *S. putrefaciens* are resistant to IPM (MICs > 4 µg/ml). *Acinetobacter* spp. is classified as intermediate (MIC = 2 µg/ml).

**Table 3:** MIC of CIP and IPM against *Acinetobacter* spp., *K. pneumoniae, P. fluorescens* and *S. putrefaciens.*

	**MIC (µg/ml)**	
	**CIP**	**IPM**
*Acinetobacter* spp.	44	2
*K. pneumoniae*	60	6
*P. fluorescens*	6	6
*S. putrefaciens*	24	12

### Bacterial surface hydrophobicity

The four bacteria had hydrophilic surfaces ($\Delta {G}_{bwb}^{TOT}$), being their values significantly different ( *P* < 0.05) (Table 4). Also, all the bacteria were predominantly electron donors (ϒ > ϒ^+^) and *K. pneumonia* and *Acinetobacter* sp. cell surfaces had similar electron donor abilities (*P* > 0.05). For *P. fluorescens* no electron accepting character (ϒ^+^ = 0 mJ/m^2^) was observed. *S. putrefaciens* had the highest value of polar component (ϒ^*AB*^) (*P* > 0.05).

**Table 4:** Surface tension parameters (ϒ^*AB*^, ϒ^+^ and ϒ^−^); and hydrophobicity (mJ/cm^2^) $\Delta {G}_{bwb}^{TOT}$. Values are means ± SDs of two independent experiments.

	Surface tension parameters (mJ/cm^2^)			Hydrophobicity−$\Delta {G}_{bwb}^{TOT}$ (mJ/cm^2^)
	ϒ^*AB*^	ϒ^+^	ϒ^−^	
*Acinetobacter* spp.	12.6 ± 0.6	0.7 ± 0.1	54.4 ± 0.6	33.7 ± 1.0
*K. pneumoniae*	19.3 ± 0.4	1.8 ± 0.1	52.1 ± 0.2	28.1 ± 0.2
*P. fluorescens*	0.0 ± 0.0	0.0 ± 0.0	69.8 ± 0.1	60.2 ± 0.1
*S. putrefaciens*	26 ± 0.6	3.7 ± 0.0	47.9 ± 0.0	22.4 ± 0.0

### Initial monolayer adhesion

**Figure 1:**
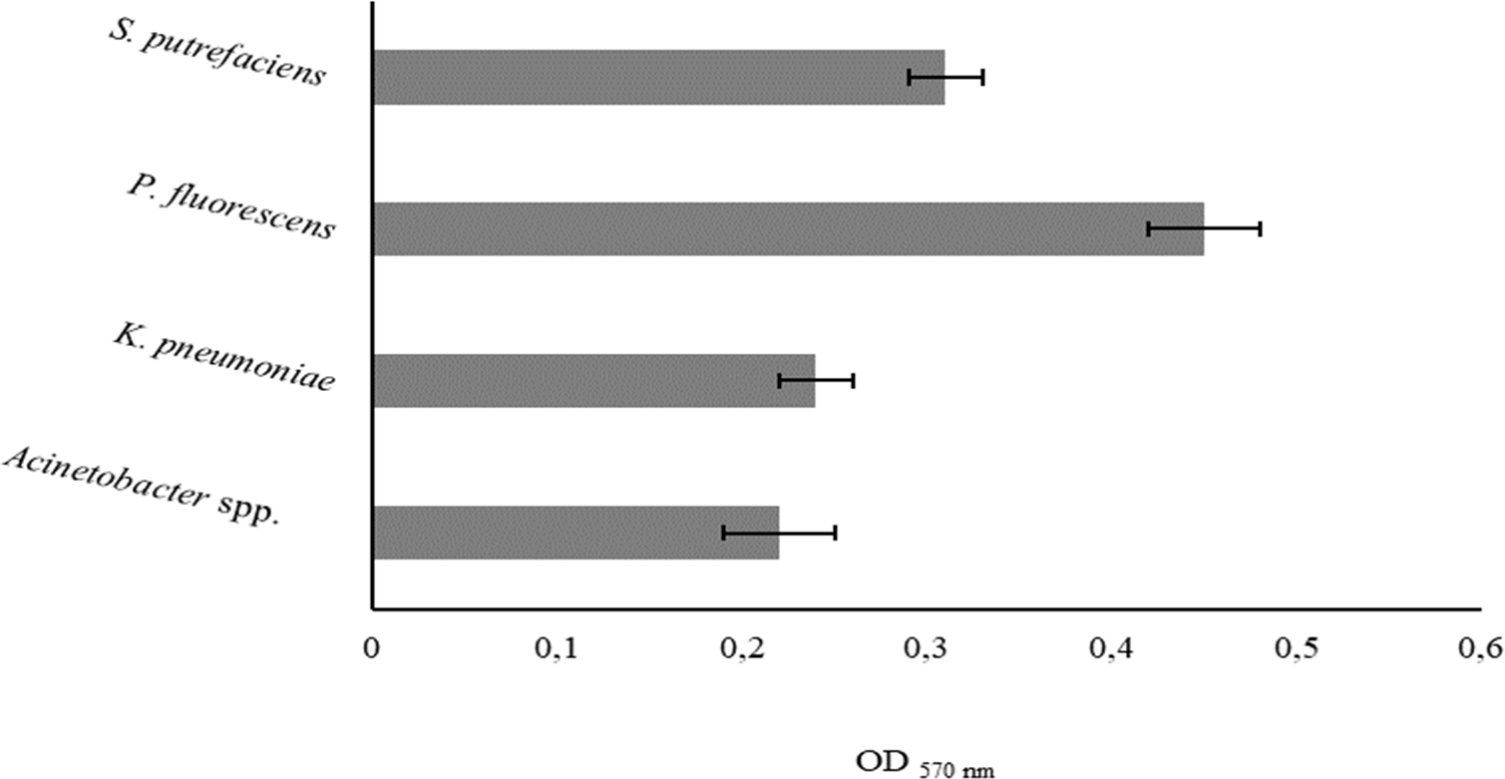
Values of OD_570 nm_ as a measure of bacterial adhesion on PS during 2 h. Mean values ± SDs for three independent experiments are illustrated.

The degree of bacterial attachment was found to follow the sequence: *P. fluorescens* > *S. putrefaciens* > *K. pneumoniae* > *Acinetobacter* spp. (Fig. 1). *S. putrefaciens*, *K. pneumoniae* and *Acinetobacter* spp. were classified as moderately adherent while *P. fluorescens* was classified as strongly adherent (Table 5). *P. fluorescens* and *Acinetobacter* spp. had the highest and lowest adhesion abilities, respectively. *K. pneumoniae* and *Acinetobacter* spp. adhered with comparable extents (*P* > 0.05).

**Table 5:** Adhesion and biofilm formation abilities of *Acinetobacter* spp., *K. pneumoniae*, *P. fluorescens* and *S. putrefaciens* according to the classification proposed by Stepanović et al. (2000).

	**Adhesion**	**Biofilm**		
		**24 h**	**48 h**	**72 h**
*Acinetobacter* spp*.*	+ +	+ +	+ +	+ +
*K. pneumoniae*	+ +	+ +	+ + +	+ + +
*P. fluorescens*	+ + +	+	+	+
*S. putrefaciens*	+ +	+	+ +	+ +

**Notes.**

(0) Non-adherent/non-biofilm producer (+) weakly adherent/weak biofilm producer (++) moderately adherent/moderate biofilm producer (+++) strongly adherent/strong biofilm producer

### Biofilm formation

**Figure 2:**
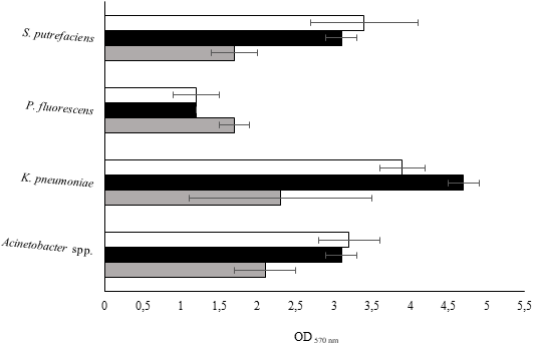
Values of OD_570 nm_ as a measure of biofilm mass at 24 (grey), 48 (black) and 72 h (white). Mean values ± SDs for three independent experiments are illustrated.

The isolates were studied for their biofilm-forming ability in 96-well polystyrene microtiter plates during 24, 48 and 72 h. Figure 2 shows that all bacteria were able to form biofilms in all the sampling times. *P. fluorescens* produced the smallest biomass amount for the three sampling times, while on the other hand *K. pneumoniae* produced the highest biomass amount. Biofilm production was directly proportional to the biofilm age for *Acinetobacter* spp. and *S. putrefaciens*. Regarding *P. fluorescens,* the biomass decreased from 24 to 48 h and remained constant for the 72 h old biofilms.

*K. pneumoniae* developed weak biofilms during the first 24 h. The biofilm formation ability increased at 48 h and decreased at 72 h. It was found that the degree of biofilm formation followed the sequence: 24 h old biofilms—*K. pneumoniae* > *Acinetobacter* spp. > *S. putrefaciens* = *P. fluorescens*; 48 h old biofilms *K. pneumoniae* > *Acinetobacter* spp. = *S. putrefaciens* > *P. fluorescens* and 72 h old biofilms—*K. pneumoniae* > *Acinetobacter* spp. = *S. putrefaciens* = *P. fluorescens*. According to the classification proposed by Stepanović et al. (2000) concerning the bacterial biofilm production ability (Table 5), *P. fluorescens* and *Acinetobacter* spp. showed weak and moderate biofilm production for the various sampling times, respectively. *S. putrefaciens* presented weak biofilm formation ability at 24 h and moderate biofilm formation ability at 48 and 72 h. *K. pneumoniae* demonstrated moderate ability to form biofilms at 24 h and strong biofilm ability at 48 and 72 h.

### Effect of ciprofloxacin and imipenem on biofilm removal

**Figure 3:**
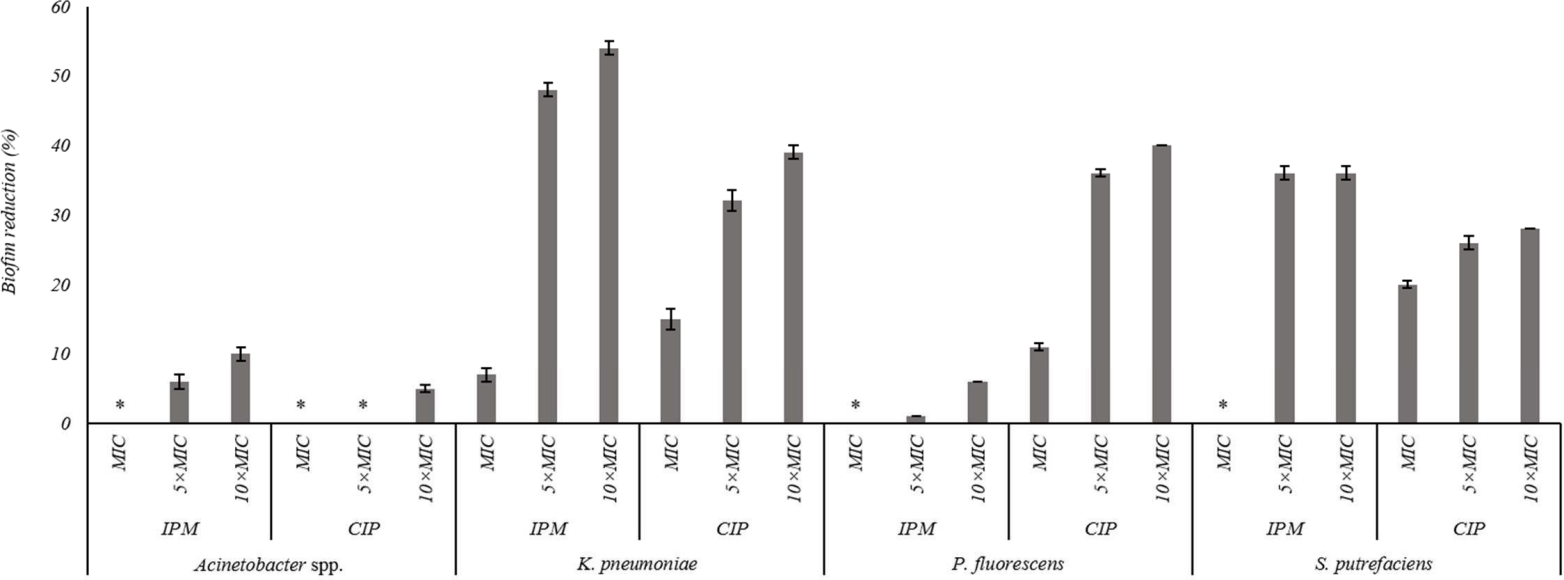
Percentage of mass reduction of 24 h old biofilms of *Acinetobacter* spp., *K. pneumoniae*, *P. fluorescens* and *S. putrefaciens* treated with IPM and CIP (at MIC, 5 × MIC and 10 × MIC) for 24 h. *No biofilm mass reduction was found. Mean values ± SDs for at least three replicates are illustrated.

The effects of the IPM and CIP were tested on the removal of 24 h old biofilms of *S. putrefaciens*, *K. pneumoniae*, *P. fluorescens* and *Acinetobacter* spp. (Fig. 3). In general, the increase of CIP or IPM concentrations increased biofilm removal. A significant increase of the *P. fluorescens* biomass reduction occurred with the increase of CIP from MIC to 5 × MIC (*P* < 0.05). However, the increase to 10 × MIC did not produce significant improvement in biofilm removal (*P* > 0.05). Similar results were observed for *K. pneumoniae* biofilms when exposed to IPM. For *S. putrefaciens* the increase of CIP concentration from MIC to 5 × MIC did not increase significantly its efficiency (Fig. 3). Moreover, for this bacterium 5 × MIC and 10 × MIC caused similar biofilm mass reductions (36%). No biofilm mass removal was obtained with IPM at MIC against *S. putrefaciens*, *P. fluorescens* and *Acinetobacter* spp., and with CIP at MIC and 5 × MIC against *Acinetobacter* spp. IPM at 10 × MIC caused the highest reductions of *S. putrefaciens* (36% removal) and *K. pneumoniae* (54% removal) biofilms. CIP at 10 × MIC caused the highest biomass reductions of *P. fluorescens* biofilms (40%). Significant reduction was also observed for *K. pneumoniae* biofilms upon the application of CIP at 10 × MIC. *Acinetobacter* spp. biofilms were highly difficult to remove using CIP and IPM (a maximum removal of 10% was obtained with IPM at 10 × MIC). In fact, *Acinetobacter* spp. biofilms were the most resistant to removal by CIP while *P. fluorescens* biofilms were the most resistant to IPM (*P* < 0.05).

## Discussion

The emergence of multidrug-resistant microorganisms remains a serious global health concern (Da Costa, Loureiro & Matos, 2013). The guts of humans and animals are important reservoirs of such microorganisms (Macfarlane et al., 2004). In general, most of the microbiological studies have been focused on the gut of human and domestics/farm animals. However, the role of wild animals as a reservoir of antibiotic resistant bacteria has been acquiring attention in recent years (Son et al., 1997; Singer et al., 2003; Finley et al., 2013; Smith et al., 2014).

In the present study, multidrug-resistant bacteria (*Acinetobacter* spp., *K. pneumoniae*, *P. fluorescens* and *S. putrefaciens*) were obtained from faecal samples of wild animals of the north of Portugal. *Acinetobacter* spp. and *K. pneumoniae* are important opportunistic pathogens due to the rapid spread of resistance to most of the currently available antibiotics, particularly to carbapenems (Poirel & Nordmann, 2006; Snitkin et al., 2012). *P. fluorescens* has been regarded generally to be of low virulence and an infrequent cause of human infections. However, they have been associated with some infections such as blood transfusion-related septicemia, catheter-related bacteremia, and peritonitis in peritoneal dialysis patients (Wong et al., 2011). *S. putrefaciens* can be widely found in the nature and especially in marine environments and, in some cases, can become pathogenic for humans and produce a wide variety of clinical conditions including bacteremia (Patel et al., 2012).

The occurrence of multidrug-resistant bacteria in wild animals that live in extremes and remote environments with low contact with antimicrobials or even never exposed to them has been previously described (Son et al., 1997; Singer et al., 2003; Da Costa, Loureiro & Matos, 2013; Smith et al., 2014). For instance, Simões et al. (2012) reported the occurrence of multidrug-resistant *Escherichia coli* hosting virulence factors in faeces of Iberian wolves (*Canis lupus signatus*). The presence of multidrug-resistant bacteria in wild animals represent a public health concern due to the increased occurrence of zoonotic diseases (Taylor, Latham & Woolhouse, 2001). Moreover, antibiotic resistance and animal health are disregarded, even if antibiotics used in human and veterinary medicine are the same (Dias et al., 2014).

Resistance to multiple antibiotics was observed in the bacteria studied in the present work. Bacteria showed resistance to IPM, a β-lactam antibiotic typically used for the treatment of complex multi-resistant human infections. β-lactam antibiotics are an important group of broad-spectrum antibiotics used in both human and animal health for the treatment of bacterial infections (Li et al., 2007). The intensive use of these antibiotics has contributed to the emergence of resistant bacteria, including bacteria of animal origin.

The production of β-lactamases, a family of enzymes that hydrolyze the β-lactam ring, thereby inactivating the antibiotic molecule prior to binding with penicillin binding proteins (PBP’s), is the principal mechanism of resistance to β-lactam antibiotics. They also play a major role in intrinsic and acquired resistance of bacteria, mainly in Gram-negative (Li et al., 2007). To understand the genes conferring resistance to β-lactam antibiotics, several genetic determinants were amplified by PCR. These results revealed the presence of genes coding for β-lactamases in *P. fluorescens* and *K. pneumoniae*. In addition, it was possible to observe that the extended spectrum β-lactamase-producing (ESBL) *K. pneumoniae* carries multiple β-lactamase genes: *bla*_OXA-aer_, *bla*_TEM_ and *bla*_SHV_. This result reinforces that ESBL-mediated plasmids are capable of transporting more than one β-lactamase gene (Rottier, Ammerlaan & Bonten, 2012). ESBL as well as TEM and SHV type producing bacteria are frequently present in the gastrointestinal tract of animals (Carattoli, 2008; Rottier, Ammerlaan & Bonten, 2012) and were already detected in wild animals (Coque, Baquero & Canton, 2008; Rottier, Ammerlaan & Bonten, 2012). It is important to note that large numbers of β-lactamases and ESBL resistant to β-lactamase inhibitors have derived from TEM and SHV enzymes as a consequence of amino acid substitution in their sequences (Paterson & Bonomo, 2005). In *P. fluorescens* only the *bla*_OXA-aer_ gene was detected. In *S. putrefaciens* and *Acinetobacter* spp. the presence of the studied β-lactamase genes was not detected, which suggest that resistance to β-lactam antibiotics in these bacteria can be mediated by other mechanisms, namely the decrease of the intracellular concentration of the antibiotic as a result of poor penetration into the bacterium or the presence of efflux pumps, and the alteration of the antibiotic target by post-translational modification of the target or genetic mutation (Bradford, 2001). Overall, the results of this study support the hypothesis that wild animals constitute a reservoir for antibiotic resistance.

During the course of their evolution, bacteria have continuously evolved their metabolism and physical characteristics adapting to almost all environments (Costerton et al., 1995). In order to survive in hostile environments bacteria have adapted to exist as communities of sessile cells (Mah & O’Toole, 2001). A direct relationship between the occurrence of microorganisms in sessile communities and infectious diseases has been reported in animals (De la Fuente-Nunez et al., 2013). According to studies conducted by Bakker et al. (2004), bacterial pathogens (*Marinobacter hydrocarbonoclasticus* ATCC 27132, *Halomonas pacifica* ATCC 27122, *Psychrobacter* sp. strain SW5H, *Staphylococcus epidermidis* GB 9/6, *A. baumannii* 2 and *P. aeruginosa*) obtained from two different niches (medical and marine) exhibited different abilities of adhesion to a surface. They concluded that this behaviour can be related to external factors like substrata, nutrients, ionic strength, pH values and temperature but also to the presence of different cell structural components (e.g., fibrils, fimbriae and adhesive surface proteins). Therefore, depending on the environmental conditions bacteria from a given niche can use different mechanisms for adhesion and biofilm formation.

Despite the central role of bacterial biofilms in infectious diseases, they are usually neglected and studies are mostly developed with cells in planktonic state. With the present work, new data is provided on the ability of bacteria isolated from wild animals to form biofilms. All the bacteria adhered on PS and produced biofilms. However, with distinct magnitudes. *P. fluorescens* was the microorganism showing the highest adhesion, but demonstrated low ability to form biofilm. *K. pneumoniae* had the highest ability to form biofilms, was the most resistant to CIP and carried multiple types of β-lactamase genes. It has been described that *K. pneumoniae* can form biofilms in the gastrointestinal tract and natural cavities of humans (Chen et al., 2014).

Surface hydrophobicity is recognized as relevant for microbial adhesion (Donlan, 2002). Liu et al. (2004) studied the role of hydrophobic and hydrophilic interactions in microbial adhesion to a surface and verified that hydrophobicity is directly related to the degree of surface adhesion. In the present study, no correlation between hydrophobicity and adhesion was found. This was particularly evident for *P. fluorescens*. This bacterium had the highest adhesion ability and the highest hydrophilic character. This result proposes that other factors, in addition to the physico-chemical surface properties, account for the adhesion process. These include the presence of extracellular structures (e.g., pili, flagella and outer membrane proteins) and genetic factors (Donlan & Costerton, 2002; Pawar, Rossman & Chen, 2005). Hennequin et al. (2012), study the resistance to antibiotics and plasmid transfer capability of clinical *K. pneumoniae* isolates in both planktonic and biofilm state. They stated the influence of capsule and ESBLs encoding plasmids upon *K. pneumoniae* adhesion. Other work developed by Yang & Zhang (2008) with *K. pneumoniae* isolated from sputum and urine demonstrated the existence of a relationship between the ability to form biofilms and the production of ESBL. These observations corroborate the results obtained in the present study, as the bacterium that encoded more β-lactamase genes was the most proficient biofilm producer. Pavlickova et al. (2017) studied the correlation between antibiotic resistance, virulence factors and biofilm formation in *E. coli* strains isolated from chicken meat and wildlife. They found a significant association between the occurrence of antibiotic resistance and the ability to form biofilm, i.e., highest prevalence of antibiotic resistance was verified in weak biofilm producers. Recent evidences on the ability of bacteria isolated from wild animals to form biofilms and the data supporting the correlation of such sessile behaviour with development of antibiotic resistance aware for the putative public health hazard of microorganisms inhabiting wild animals (Schillaci & Vitale, 2012).

Microorganisms in biofilms are more resistant to the action of the antimicrobial agents and host defence mechanisms due to several mechanisms, particularly the presence of a matrix of extracellular polymeric substances, low growth rate, presence of persister cells and the expression of possible biofilms specific resistance genes (Stewart & Costerton, 2001; Stewart, 2002). Biofilms are also important in the spread of antibiotic resistance by horizontal genes transfer (Fux et al., 2005). In the present study the effects of CIP and IPM (applied at MIC, 5 × MIC and 10 × MIC) on biofilm removal were evaluated. IPM and CIP are antibiotics commonly used to control bacterial infections and present wide spectrum of activity (Tamma, Cosgrove & Maragakis, 2012). In general, a dose-dependent effect was observed and the maximum percentage of biofilm mass reduction was <55%. For two of the tested bacteria (*P. fluorescens* and *Acinetobacter* spp.) both antibiotics caused no biomass removal at MIC. *Acinetobacter* spp. and *P. fluorescens* biofilms were also the most difficult to remove by CIP and IPM, respectively. No reports are available on the action of the selected antibiotics against biofilms formed by bacteria isolated from animals or other unexploited environmental source. However, high levels of resistance to CIP and/or IPM has been reported among biofilms of clinical strains of *A. baumannii* and *P. aeruginosa* (Abdi-Ali et al., 2014; Hengzhuang et al., 2011). In the present study, IPM was the most effective in *S. putrefaciens* and *K. pneumoniae* biofilm mass removal, however, total removal was not achieved, which is a critical aspect in biofilm control. In fact, antibiotics can promote significant killing of biofilm cells without causing their dispersal/removal (Roizman et al., 2017).

## Conclusions

In conclusion, the existing levels of resistance to antibiotic in wild animals can create a continuous selective pressure on β-lactamases, which is a huge concern to public and animal health. The analysis of the phenotypic and genotypic resistance profile of the bacteria studied in this work demonstrated their multidrug-resistant character, and some of them possess antibiotic resistance determinants. Taking into account that wildlife isolates do not contact directly with antibiotics, the resistance observed among the studied bacteria is alarming. This is mainly related to the overuse of antibiotics not only in human but also in veterinary medicine, leading to the spread of resistance genes to the environment. The selected bacteria also demonstrated the ability to form biofilms on PS surfaces, and further tests revealed the inefficacy of two broad-spectrum antibiotics, CIP and IPM, to induce strong biofilm dispersal. Biofilm formation was independent on the bacterial surface properties.

The overall study proposes that regular monitoring of multidrug-resistance and proper characterization and assessment of the antimicrobial resistance determinants among bacteria of animal origin could prevent the dissemination of antibiotic resistant microorganisms and genetic determinants containing antibiotic resistance genes.

##  Supplemental Information

10.7717/peerj.4974/supp-1 Figure S1 Unrooted phylogenetic tree based on 16S rRNA gene sequences of *Acinetobacter* spp. isolated in this study and the bacteria of the Nacional Center forBiotechnology Information (NCBI) databases

10.7717/peerj.4974/supp-2 Supplemental Information 1 Raw data from adhesion experiments

10.7717/peerj.4974/supp-3 Supplemental Information 2 Raw data from biofilm formation experiments

10.7717/peerj.4974/supp-4 Supplemental Information 3 Raw data from contact angle measurements

10.7717/peerj.4974/supp-5 Supplemental Information 4 Raw data from biofilm control with antibiotics—CIP_IPM

## Additional Information and Declarations

### Field Study Permissions

The following information was supplied relating to field study approvals (i.e., approving body and any reference numbers): The Centre for Treatment of Wild Animals (University of Trás-os-Montes and Alto Douro) approved the field experiments under the PhD project SFRH/BD/90120/2012.

### Data Availability

The following information was supplied regarding data availability: The raw data are provided as Supplemental Files.
