# Supplementary figures and images for: Biofilms and antibiotic susceptibility of multidrug-resistant bacteria from wild animals
Source: PeerJ. 2018 Jun 12;6:e4974. doi: 10.7717/peerj.4974 (PMC6003395; doi:10.7717/peerj.4974)

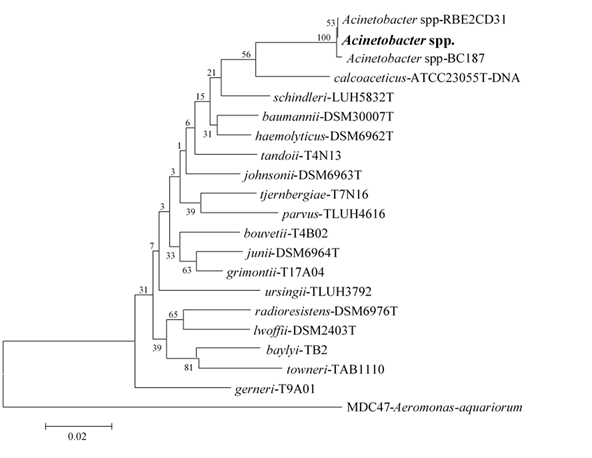

Supplement: Figure S1 [file peerj-06-4974-s001.png]
